# Early immune profiling reveals distinct inflammatory responses between children and adults few days after primary SARS-CoV-2 infection

**DOI:** 10.3389/fimmu.2024.1359993

**Published:** 2024-11-18

**Authors:** Martijn D. B. Van de Garde, Alberto Miranda-Bedate, Nening M. Nanlohy, Ronald H. J. Jacobi, Adam Meijer, Daphne F. M. Reukers, Josine Van Beek, Cecile A. C. M. Van Els, Debbie Van Baarle, Nynke Y. Rots, Jelle De Wit, Elena Pinelli

**Affiliations:** ^1^ Centre for Infectious Disease Control, National Institute for Public Health and the Environment (RIVM), Bilthoven, Netherlands; ^2^ Infectious Diseases and Immunology, Department of Biomolecular Health Sciences, Faculty of Veterinary Medicine, Utrecht University, Utrecht, Netherlands; ^3^ Department of Medical Microbiology and Infection Prevention, Virology and Immunology Research Group, University Medical Center Groningen, Groningen, Netherlands

**Keywords:** COVID-19, innate immune response, age, monocytes, toll like receptors

## Abstract

**Background:**

To date, it is still not clear why during the COVID-19 pandemic children generally developed no or milder symptoms compared to adults. As innate immune responses are crucial in the early defense against pathogens, we aimed at profiling these responses from both adults and children with a primary SARS-CoV-2 infection.

**Methods:**

In the first months of the pandemic, PBMCs and serum were collected from peripheral blood of adults and children at different time points after testing SARS-CoV-2 PCR positive (PCR+). The levels of SARS-CoV-2 Spike-specific IgG were measured in serum. The cells were cultured for 24 hours in medium only, with heat inactivated SARS-CoV-2 (iSARS-CoV-2) or toll-like receptor (TLR) ligands. The levels of secreted cytokines/chemokines as well as monocyte phenotype were determined.

**Results:**

Few days after testing PCR+, PBMCs from PCR+ children secreted higher levels of cytokines/chemokines compared to PCR+ adults, after these cells were incubated either in medium only or after stimulation with iSARS-CoV-2 or TLR ligands. Furthermore, PBMCs from children stimulated with iSARS-CoV-2 secreted significantly higher levels of IL-10 and GM-CSF compared to PBMCs from control children. In contrast, PBMCs from the PCR+ adults secreted lower levels of IL-8 compared to adult controls. Phenotypic analysis of monocytes indicates a smaller proportion non-classical monocytes for adults compared to children. The distinct cytokine profiles, symptom severity, and the proportion of non-classical monocytes correlated to each other. The levels of Spike-specific IgG overtime did not significantly differ between children and adults.

**Conclusions:**

Within the first week after testing PCR+, children showed a stronger inflammatory innate immune profile and experienced less severe symptoms compared to adults. Our data implies correlations between the secretion of cytokines/chemokines, proportion of non-classical monocytes, and symptoms severity. These findings enhance our understanding of the distinct pediatric and adult innate immune profile after SARS-CoV-2 infection and contributes to the knowledge necessary to improve future prevention strategies.

## Introduction

Towards the end of 2019, a novel coronavirus, designated as SARS-CoV-2, emerged in Wuhan, China, causing coronavirus disease-2019 (COVID-19) which spread worldwide and was declared in March 2020 by the WHO as a global pandemic (1, 2). Millions of cases and deaths were reported globally and different control and prevention measures were introduced (3). At present COVID-19 no longer represents a global health emergency, however, due to cases with long-term symptoms as well as breakthrough infections and the decline of vaccine-induced immunity, it still remains crucial to better understand the immune response to infection with this virus.

One of the remarkable aspects during the COVID-19 pandemic was that the symptoms were generally less frequent in children and if any, these were milder compared to those in adults (4–6). Although it is not fully understood why there are differences in the clinical manifestations between adults and children, several studies point towards the crucial role of the antiviral-immune response early after SARS-CoV-2 infection (7–10). Several PRRs, like the endosomal Toll-like-receptors (TLR) (TLR7 and TLR8) and RIG-I like receptors (RLR) (RIG-I and MDA5), can be triggered by SARS-CoV-2 RNA (11). Extracellular TLRs, usually recognize bacterial lipoproteins and lipopolysaccharides, respectively, although different viral proteins, like the SARS-CoV-2 spike protein, have been shown to activate these PRRs as well (8). Epithelial and immune cells from children have higher cell-intrinsic antiviral capacity and express higher levels of various PRRs and are therefore posed to be better primed for viral sensing, compared to adults (9, 10, 12). Age intrinsic differences in susceptibility to SARS-CoV-2 were also demonstrated using bronchial epithelial cell cultures form children and adults, showing a much more attenuated inflammatory response to SARS-CoV-2 by cells from children (13). The exact innate immune mechanisms that limit SARS-CoV-2 infection and COVID-19 symptoms remains to be determined.

The aim of this study was to determine differences between the innate immune profiles from children and adults with a SARS-CoV-2 infection and if these correlated with symptom severity and whether these profiles changed over time. To this end, peripheral blood mononuclear cells (PBMCs) and serum samples were collected from parents and children from the same household that had tested PCR positive for SARS-CoV-2 (PCR+) early in the pandemic. Next to the development of SARS-CoV-2 specific antibodies, the cytokine and chemokine secretion profiles along with the phenotype of monocytes upon ex-vivo stimulation with heat-inactivated SARS-CoV-2 (iSARS-CoV-2) and different TLR ligands were examined.

## Materials and methods

### Ethical statement

Samples used in this study derive from three different studies and were all approved by the Medical-Ethical Review Committee of the University Medical Center Utrecht (SARS-CoV-2 transmission study: NL13529.041.06, NVI-255 study: NL29241.000.09, and IMMFACT study: NL46795.094.13). Written informed consent was obtained from all participants and all trial-related activities were conducted according to Good Clinical Practice, including the provisions of the Declaration of Helsinki.

### Population and sample collection

Participants of the previously described SARS-CoV-2 transmission study (14) included persons 18 years and older with a positive SARS-CoV-2 PCR test who had at least one child in their household. All household contacts (adults and children) were enrolled in the study, except for contacts below one year of age. This study was carried out in the Netherlands early in the COVID-19 pandemic in the period of March-April 2020. During home visits blood samples were collected for serum and PBMC isolation. In order to study the early immune responses, index case participants were selected with a first blood collection home visit (T1), 3 to 7 days, after testing PCR+ during routine COVID19 surveillance (**Table 1**). At time the index case participant was sampled, their household members also provided samples. Household members who tested PCR+ in either nose, throat, saliva, or feces were included in this study. The two follow-up time points took place 2-3 weeks (T2), 4-6 weeks (T3) after T1. For children, no PBMCs were collected at T3. PBMCS from healthy adults (NVI-255 study) (15) and healthy children (IMMFACT study) (16), both collected several years before the COVID-19 pandemic, were also included as SARS-CoV-2 unexposed controls.

**Table 1:** Demographic and clinical characteristics of unexposed healthy controls and the PCR confirmed participants with a SARS-CoV-2 infection.

Characteristics	PCR+	HC*	PCR+	HC*
	Children	Children	Adults	Adults
	N = 24	N = 13	N = 21	N = 12
Median age (range) – years	11.5 (2 – 16)	11 (2 – 16)	41.1 (19 – 71)	36.8 (25 – 51)
Sex – number (%)				
Male	11 (45.8)	4 (31.8)	9 (43)	6 (50.0)
Female	13 (54.2)	9 (69.2)	12 (57)	6 (50.0)
PCR positivity – number (%)				
Overall^a^	24 (100.0)	N/A	21 (100.0)	N/A
Nasopharyngeal swabs	21 (87.5)		16 (76)	
Saliva	10 (41.7)		12 (57)	
Feces	20 (83.3)		15 (71)	
Disease severity – number (%)				
Asymptomatic	3 (12.5)	N/A	0 (0)	N/A
Mild	18 (75)		11 (52)	
Moderate	3 (12.5)		7 (33)	
Severe	0 (0)		3 (14)	

^a^At least one positive PCR in one source; N/A, Not applicable; *Healthy Controls from before the COVID-19 pandemic.

### Symptoms and severity of COVID-19

As previously described (14) severity of COVID-19 was classified into mild, moderate and severe, which were indicated by severity indexes 1, 2 and 3, respectively. Symptoms reported by participants included sore throat, cough, respiratory difficulties, fever, chills, headache, muscle pain, joint ache, diarrhea, nausea, vomiting, loss of appetite or fatigue. Participants with mild disease presented any of the clinical symptoms described above. Those with moderate COVID-19 reported having pneumonia, including dyspnea, and those with severe disease reported dyspnea and have consulted a healthcare professional or have been admitted to the hospital. The overall disease severity for each participant was calculated, by adding the severity indexes of each participant during the first three home visits which resulted in: No Symptoms (0), Mild (1-2) Moderate (3-4) and Severe disease (>5).

### Inactivated SARS-CoV-2 virus preparation

A SARS-CoV-2 heat-inactivated viral suspension (iSARS-CoV-2) was used in the present study. The SARS-CoV-2 isolate (hCoV-19/Netherlands/ZuidHolland_10004/2020) was obtained from a Dutch patient (Centre for Infectious Disease Control, RIVM). The virus was grown on VERO-E6 cells as previously described (17). Heat-inactivation was performed by incubating the virus at 60°C for 2 hours after which the samples were stored at −80°C.

### PBMCS isolation, stimulation, cytokine determination and flow cytometry

PBMCS were isolated from whole blood using Ficoll–Paque density centrifugation and cryopreserved following standardized protocols. Thawed PBMCS (2x10^5^ cells/well) were stimulated for 24 h in the presence of inactivated (i)SARS-CoV-2 (MOI=3) or different PRR-ligands: 10^7^ cells/mL heat killed *Listeria monocytogenes* (HKLM, TLR2 ligand), 2.5 mg/mL R848 (TLR7/8 ligand) or 25 ng/mL Lipopolysaccharide (TLR4 ligand) (Invivogen).

After 24 h, supernatants were collected and analyzed for cytokine production by Legendplex, measuring IL-1β, IL-6, TNFα, CXCL10, IFNλ1, IL-8, IFNα2, GM-CSF, IFNβ, IL-10, IFNγ, IL-12-p70, IFNλ2/3 (LEGENDplex™ Human anti-Virus response Panel (13-plex), BioLegend) following manufacturer’s instructions. IL-12-p70 levels were below lower limit of detection and were therefore excluded from further analysis.

After collection of the supernatants, the stimulated cells were analyzed for different monocyte subset markers by flow cytometry. For flow cytometry, cells were washed with 0.5%BSA/PBS and incubated 5 min with Trustain (10x in 0.5%BSA/PBS, Biolegend), thereafter a mixture of antibodies (all from Biolegend, unless stated otherwise) was added for 20 min: CD14-FITC (clone HCD14), CD16-APC (clone 3G8), CD3-AlexaFluor700 (clone HIT3a), CD4-AlexaFluor700 (clone RPA-T4), CD19-AlexaFluor700 (clone HIB19), CD56-AlexaFluor700 (clone HCD56), CD15-AlexaFluor700 (clone HI98), NKp56-AlexaFluor700 clone 9E2), Fixable Viability Stain780 (BD Biosciences), HLA-DR-BrilliantViolet510 (clone L243), CCR5-BrilliantViolet421 (clone J418F1), CCR2-BrilliantViolet605 (clone 036C2), CX3CR1-PE/Dazzle594 (clone 2A9-1), CD11c-PE (clone 3.9) and CD11b-BrilliantUV737 (clone M1/70, BD Biosciences). After washing, the cells were acquired on a FACS Symphony (BD) and total monocytes were gated using FlowJo Software (V10.6.2). To identify the specific monocyte populations, preprocessing and clustering of the FACS datasets was performed in R v4.2.0 (18) and RStudio (v2022) with packages flowCore (19) and CATALYST (20, 21) (**Supplementary Figure 1**).

### SARS-CoV-2 multiplex immunoassay

SARS-CoV-2 multiplex immunoassays (MIA) were carried out to determine specific serum antibody IgG levels towards SARS-CoV-2 Spike S1 (Sino Biological, 40591-V08H) as described previously (22). In short, the serum was incubated with antigen-coupled beads for 45 minutes in the dark, followed by a 30-minute incubation with Goat-anti-human IgG. Samples were incubated in SM01 (Surmodics) and washing steps after each incubation were carried out with PBS. Antibody binding to antigen-coupled beads was determined with FM3D (Luminex) and antibody levels were expressed as binding antibody units (BAU)/ml for S1. The threshold for seropositivity was set at 10.1 BAU/mL for Spike S1, as previously standardized for the Wuhan (vaccine) strain against the NIBSC/WHO COVID-19 reference serum 20/136 (23).

### Statistical analysis

Statistical analysis was performed using non-parametric Kruskal-Wallis test for unpaired analysis or Friedmans test for paired samples over time, both followed by Dunn’s multiple comparison test, or multiple Mann-Whitney test with Bonferroni-Dunn multiple comparison test, as indicated, all in Graphpad Prism version 9.5.1. Differences between groups were considered statistically significant at adjusted p-value <0.05. Spearman’s correlation analysis was used between cytokine levels and the proportion of monocyte subsets, or disease severity. A correlation was considered significant at adjusted p-value <0.05. Kruskal-Wallis Chi-squared test was used to analyze proportion of monocyte subsets with disease severity, p-value <0.05 was considered significant.

## Results

### PBMCs from PCR+ children stimulated with inactivated SARS-CoV-2 secrete higher levels of cytokines and chemokines compared to adults

**Figure 1:**
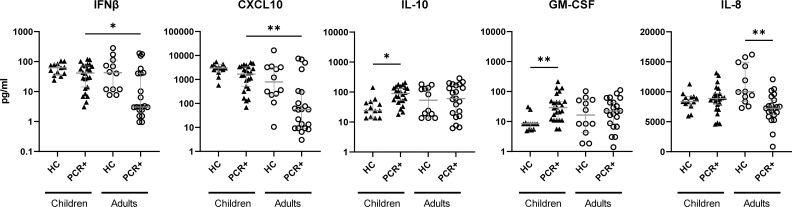
Divergent cytokine and chemokine profile induced by iSARS-CoV-2 in PBMCs from healthy control and recently infected children and adults. Cytokine and chemokine levels were measured in culture supernatant from PBMCs after overnight stimulation with iSARS-CoV-2 (MOI=3). Kruskal-Wallis non-parametric test was used to compare healthy control (HC) to PCR+ individuals among children (▲) or adults (○), as well as HC or PCR+ children to their counterparts in adults, after which Dunn’s multiple comparison test was executed. Error bars indicate median value with 95% CI. *p-adj<0.05. **p-adj-<0.01.

PBCMs taken from children and adults within the first week after testing PCR+ for SARS-CoV-2 early in the pandemic were stimulated *in vitro* with the iSARS-CoV-2 preparation and the cytokine levels were measured 24 hours after incubation. PBMCs from the PCR+ children secreted significantly higher levels of IFNβ and CXCL10 compared to the PBMCs from PCR+ adults, after iSARS-CoV-2 stimulation (**Figure 1**). Compared to the corresponding unexposed healthy controls, stimulated PBMCs from the PCR+ children produced significant higher levels of IL-10 and GM-CSF (**Figure 1**). In contrast, PBMCs from the PCR+ adults secreted lower levels of IL-8 compared those from adult healthy controls (**Figure 1**). No significant differences were observed between children and adults in the healthy control groups. These data indicate different innate immune responses between PCR+ children and adults as well as between age-matched PCR+ and healthy individuals.

### The levels of cytokines and chemokines secreted after iSARS-CoV2 stimulation do not correlate with the Spike-specific IgG antibodies dynamics

**Figure 2:**
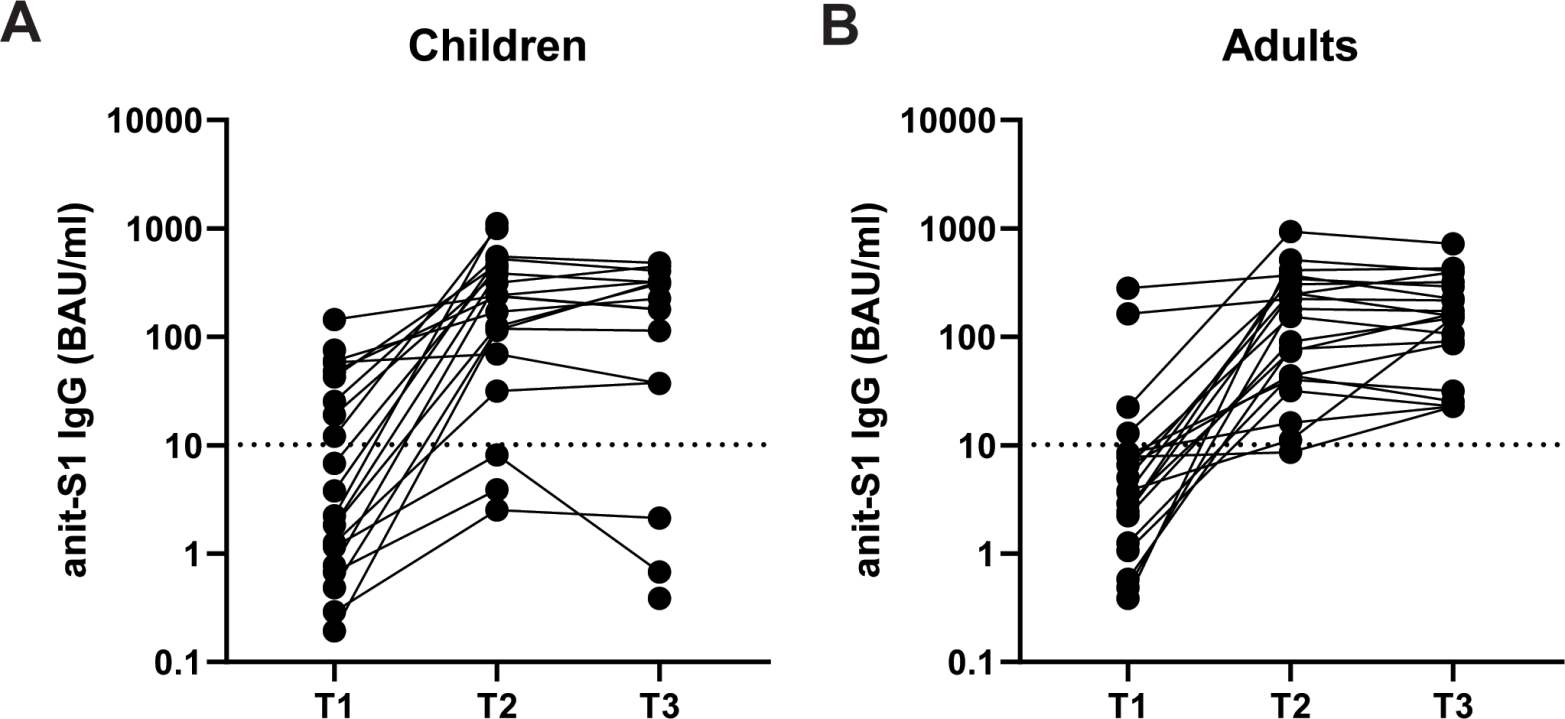
The development of anti-SARS-CoV-2 Spike-specific antibodies over time in PCR+ children and adults. SARS-CoV-2 Spike-specific IgG levels were measured within <7 days (T1), 2-3 weeks (T2), 4-6 weeks (T3) after testing PCR+ in serum of children **(A)** and adults **(B)**. IgG levels are expressed as binding antibody units (BAU)/ml. Dotted line indicates seropositivity threshold of 10.1 BAU/ml.

The development of specific antibody response after primary SARS-CoV-2 infection was measured at the different time points <7 days (T1), 2-3 weeks (T2) and 4-6 weeks (T3) after testing PCR+. For 22/24 children there were T2 and/or T3 serum sample available to determine SARS-CoV-2 Spike-specific IgG antibody levels. Four out of the 22 children did not show Spike-specific IgG levels at T2 and/or T3 (**Figure 2A**), whereas all the adults did develop Spike-specific IgG antibodies at T3 (**Figure 2B**). The Spike-specific antibody levels did not significantly differ between children and adults at any given timepoint. Next, we assessed whether the innate responsiveness of PBMCs at T1 stimulated with iSARS-CoV-2 could predict the development of the spike-specific IgG titers at T2 and T3. However, we did not find any significant association between the levels of cytokines and chemokines secreted by PBMCs after iSARS-CoV-2 stimulation and the Spike-specific IgG levels at T2 and T3.

### The levels of IL-10, GM-CSF and IL-8 secreted by PBMCs after iSARS-CoV-2 stimulation correlate with symptom severity

**Figure 3:**
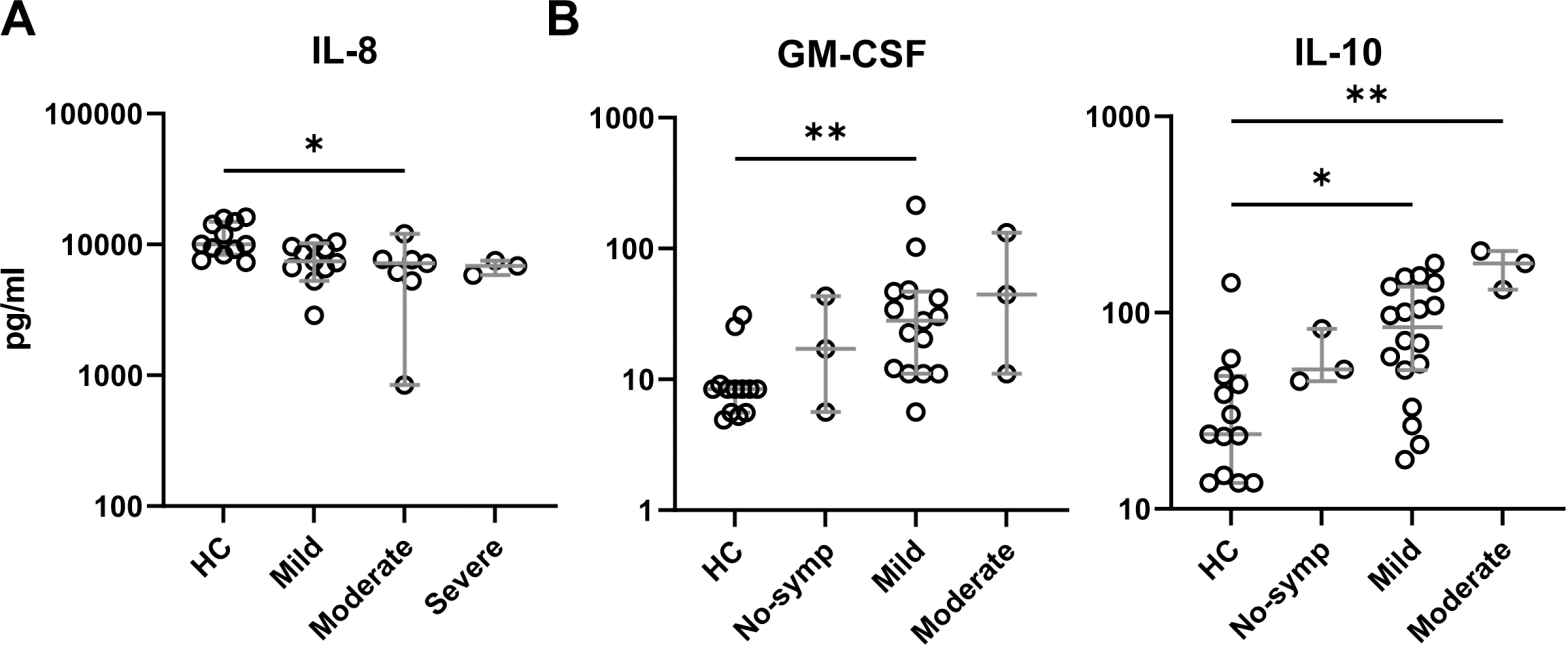
Symptom severity correlates with cytokine/chemokine production. Cytokine and chemokine levels delineated by symptom severity groups in adults **(A)** and children **(B)**. Kruskal-Wallis non-parametric with Dunn’s multiple comparison test was performed comparing each group to each other. Error bars indicate median value with 95% CI. *p-adj<0.05. **p-adj<0.01.

Since the levels of some of the cytokine and chemokines secreted by PBMCs from PCR+ adults and children were highly variable after iSARS-CoV-2 stimulation, we questioned whether this could be related to the clinical status of the participants. Indeed, correlation analysis indicated a relation between the levels of IL-8 and the clinical status in adults (r=-0,527 p=0.0016). In children the levels of GM-CSF and IL-10 correlated with symptom severity (r=0.466, p=0.0036; r=0.621, p<0.001, respectively). Compared to healthy adults, the levels of IL-8 were significantly lower for adults with moderate symptoms (**Figure 3A**). No significant correlation was found between the levels of IFN-β or CXCL10 and symptom severity for adults (data not shown). For PBMCs from children with mild symptoms significantly higher levels of GM-CSF and IL-10 were found to be secreted after iSARS-CoV-2 stimulation, compared to their healthy counterparts (**Figure 3B**). Furthermore, the levels of IL-10 were also significantly higher for children with moderate symptoms (**Figure 3B**). These findings indicate distinct associations between cytokine responses and symptom severity in children and adults.

### Monocyte subset proportions differ between PCR+ adults and children, and associate with cytokine responses

**Figure 4:**
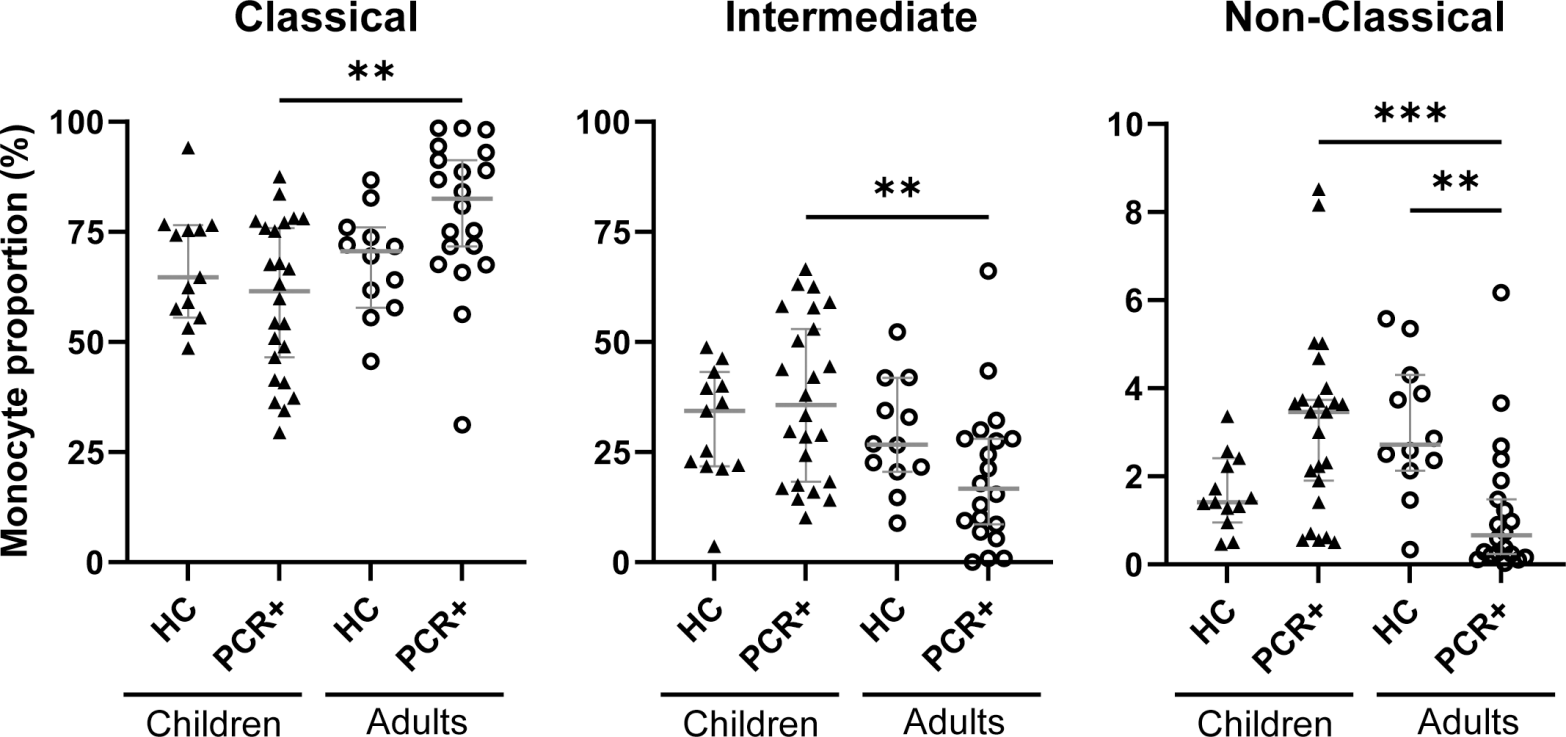
Monocyte subset proportions differ between PCR+ children and adults. Flow cytometric analysis of monocytes within PBMCS after iSARS-CoV-2 stimulation. Proportions of classical monocytes (CD14+CD16-), intermediate monocytes (CD14+CD16+), and non-classical monocytes (CD14lowCD16+) among total monocytes in PBMCs. Kruskal-Wallis non-parametric test was used to compare HC to PCR+ individuals among children (▲) or adults (○), as well as HC or PCR+ children to their counterparts in adults, after which Dunn’s multiple comparison test was executed. Error bars indicate median value with 95% CI. **p-adj-<0.01. ***p-adj<0.001.

Monocytes are the most abundant PRR expressing cells in the PBMCs compartment and may determine the outcome of the PBMCs interaction with iSARS-COV-2 (24). Phenotypic analysis indicates that upon *in vitro* iSARS-CoV-2 stimulation, the classical monocytes in PBMCs of PCR+ adults were the most abundant subset, which was significantly higher compared to PBMCs from PCR+ children (**Figure 4**). In contrast, iSARS-CoV-2 stimulated PBMCs from PCR+ children presented significantly larger proportion of intermediate and non-classical monocytes compared to those in adults (**Figure 4**). In adults the proportion of non-classical monocytes was significantly smaller in PCR+ compared to healthy individuals (**Figure 4**). Over time the proportion of monocyte subsets in PCR+ adults changed to levels similar to those of healthy controls with smaller proportion of classical, and larger proportion of non-classical monocytes at T2 and T3 (**Supplementary Figure 2**).

To assess whether changes in monocyte subset proportions relate to cytokine and chemokine production after iSARS-CoV-2 stimulation early after infection (T1) we performed correlation analysis. The proportion of classical and intermediate monocytes in PBMCs from PCR+ adults showed a significant correlation with IL-1β, GM-CSF and IL-10 secretion (**Table 2**). The non-classical monocyte subset correlated with interferon (IFNλ1, IFNα2, and IFNβ), CXCL10, and IL-8 secretion. Proportion of non-classical monocytes in PBMCs from PCR+ children also showed a significant correlation with interferons (IFNα2 and IFNβ), CXCL10, and IL-8, albeit less strong as those in adults (**Table 2**). Comparing the proportion of non-classical monocyte between healthy control and PCR+ individuals we observed significantly lower values in adults, but not in children, with moderate symptoms (p-adj = 0.0088). The correlation between non-classical monocytes, symptom severity, and the production of interferons, CXCL10 and IL-8 after iSARS-CoV-2 stimulation indicates important differences in anti-viral innate immune response between PCR+ children and adults early after infection.

**Table 2:** Correlation of monocyte subsets with cytokine production in PCR+ individuals.

			IL-1β	GM-CSF	IL-10	CXCL10	IFNλ1	IL-8	IFNα2	IFNβ
Children	Classical monocytes	r2	–	–	–	–	–	–	–	–
		p	ns	ns	ns	ns	ns	ns	ns	ns
	Intermediate Monocytes	r2	-	-	-	-	-	-	-	-
		p	ns	ns	ns	ns	ns	ns	ns	ns
	Non-classical Monocytes	r2	–	–	–	0.2091	–	0.1835	0.1758	0.2179
		p	ns	ns	ns	0.0247	ns	0.0368	0.0414	0.0215
Adults	Classical monocytes	r2	0.3706	0.4146	0.4229	–	–	–	–	–
		p	0.0044	0.0022	0.0019	ns	ns	ns	ns	ns
	Intermediate Monocytes	r2	0.3686	0.4203	0.4369	-	-	-	-	-
		p	0.0045	0.0020	0.0015	ns	ns	ns	ns	ns
	Non-classical Monocytes	r2	–	–	–	0.5925	0.6227	0.3198	0.5973	0.6332
		p	ns	ns	ns	<0.0001	<0.0001	0.0094	<0.0001	<0.0001

ns, not statistically significant.

### TLR activation results in increased cytokine and chemokine secretion by PBMCS from PCR+ children compared to adults

**Figure 5:**
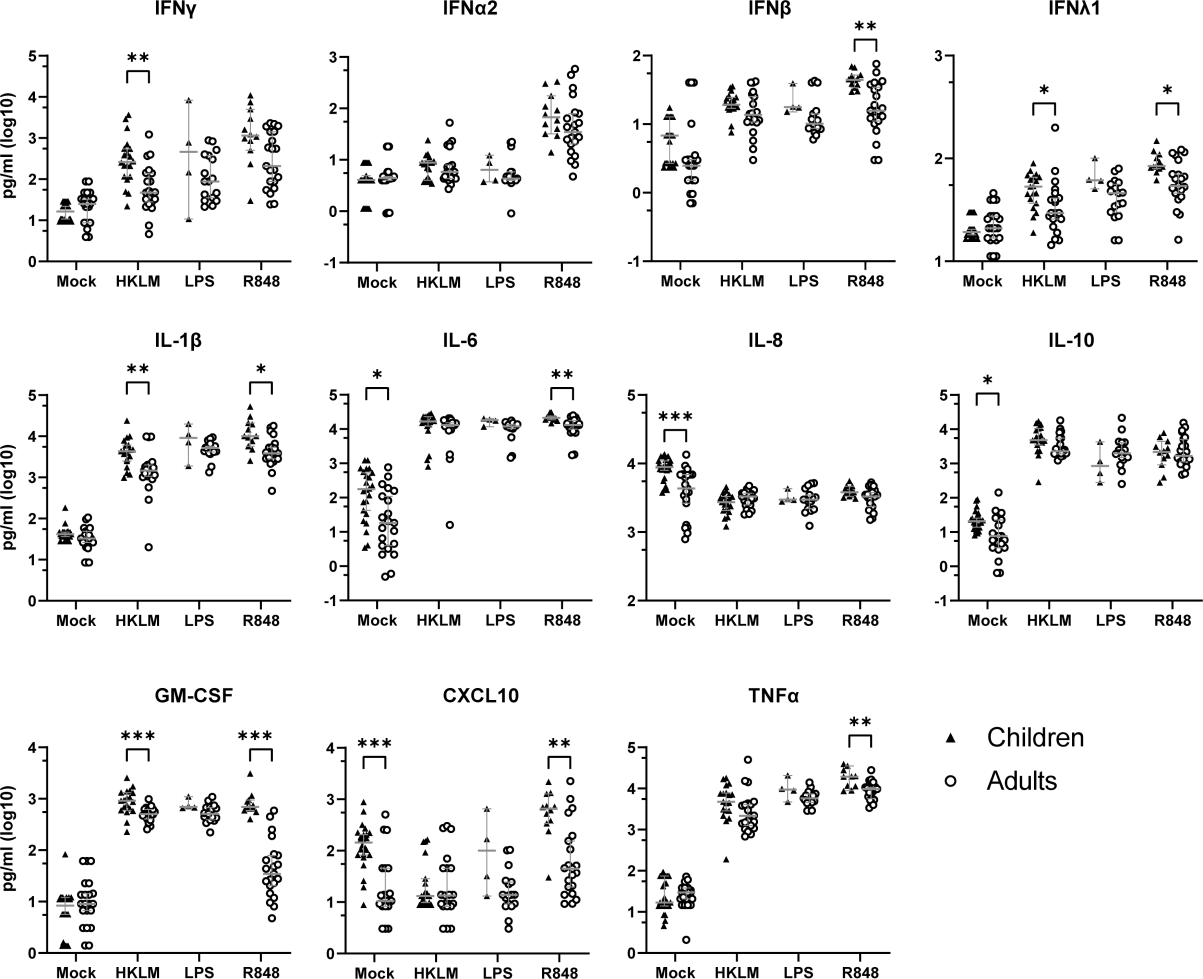
PBMCs from PCR+ children secret more cytokines and chemokines after TLR activation compared to adults. Cytokines and chemokines were measured in supernatant of PBMCs from PCR+ children (▲) and adults (○) after overnight stimulation of TLR2 with HKLM, TLR4 with lipopolysaccharide (LPS), or TLR7/8 with Resiquimod (R848). Values are shown as log10 pg/ml. Multiple Mann-Whitney test with Bonferroni-Dunn multiple comparison test were performed to compare response by children and adults for each stimulation or mock. Error bars indicate median value with 95% CI. *p-adj<0.05. **p-adj<0.01. ***p-adj<0.001.

Several studies have highlighted the importance of PRR activation including that of TLR2, TLR4 and TLR7/8 in the innate immune response to SARS-CoV-2 (25–27). Therefore, we next measured the responsiveness of PBMCS from the PCR+ children and adults stimulated with pathogen-associated molecular patterns activating these PRR. Results indicate that PBMCS from the PCR+ children secrete significantly higher levels of IL-1β, IL-6, IFNλ1, IFNβ, TNFα, CXCL10 and GM-CSF after stimulation with TLR7/8 ligand R848 compared to adults (**Figure 5**). In addition, we observed that activation of TLR2 by HKLM resulted in significantly higher levels of IL-1β, IFNλ1, IFNγ, TNFα and GM-CSF for the PBMCS from the PCR+ children (**Figure 5**). Activation of TLR4 with LPS did not result in significant differences although a trend was observed for increased levels of several of these cytokines for the PCR+ children (**Figure 5**). Interestingly, when PBMCS from the PCR+ individuals were cultured overnight in medium only (Mock) the levels of secreted IL-6, IL-8, IL-10, and specially CXCL10 were already significantly higher for PCR+ children compared to PCR+ adults (**Figure 5**). For mock-stimulated PBMCs from healthy controls, higher levels of CXCL10 and IFNβ were also observed in children, but no increased levels of IL-6, IL-10 and IL-8 were found (**Supplementary Figure 3**). Also worth noticing is that while in healthy controls the levels of IL-8 after activation with the different TLR ligands were significantly higher for adults compared to children, this difference was reversed for the PCR+ adults. Altogether, these findings indicate that TLR activation, particularly TLR7/8, shows pronounced differences in the cytokine secretion profile between PCR+ children versus adults and that mock stimulated PBMCs already show some differences in the response between these two age groups.

### Several weeks after testing SARS-CoV-2 PCR+ adults PBMCs show increased cytokine and chemokine secretion upon stimulation

**Figure 6:**
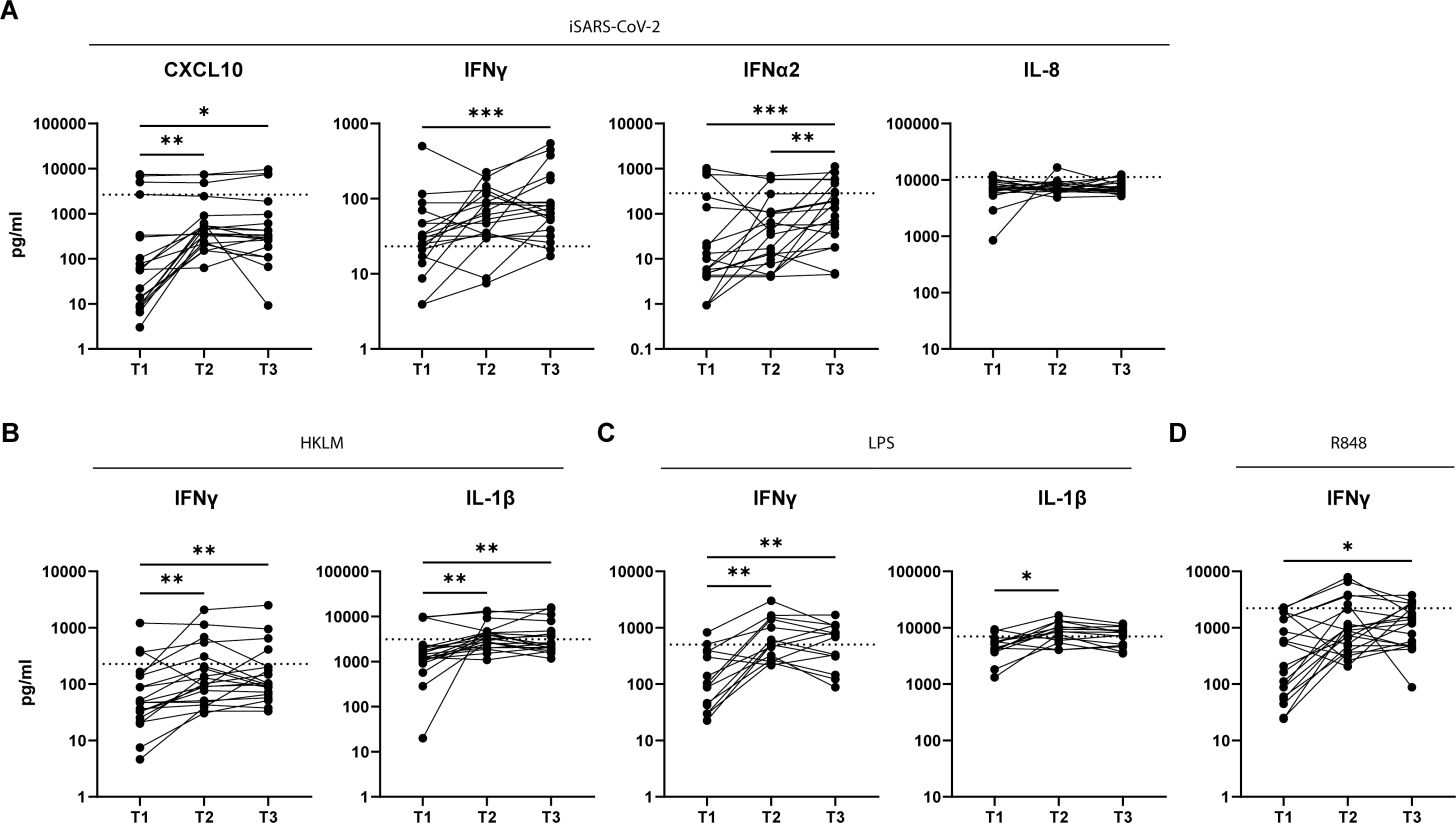
Adult PBMCs produce more cytokines and chemokines at later time points post infection. Cytokines and chemokines were measured in culture supernatant after iSARS-CoV-2 **(A)**, HKLM **(B)**, LPS **(C)**, or R848 **(D)** stimulation of adult PBMCs collected at T1 (<7days), T2 (2-3 weeks), and T3 (4-6 weeks) post infection. Dotted line indicates average value of healthy control group. Friedmans test for non-parametric paired samples. followed by Dunn’s multiple comparison test was used to compare responses at each time point. *p-adj<0.05. **p-adj<0.01. ***p-adj<0.001.

To determine whether the cytokine secretion profile observed within a few days after testing PCR positive (T1) changed in time, we tested the responsiveness to iSARS-CoV-2 by PBMCs from the same adult individuals 2-3 weeks (T2) and 4-6 weeks (T3) later. The levels of CXCL10 after iSARS-CoV-2 stimulation of adult PBMCs were significantly higher at T2 and T3 compared to T1, and the levels of IFNγ and IFNα2 were significantly higher at T3 compared to T1 (**Figure 6A**). Notably, the levels of IL-8 remained lower compared to those of healthy controls up to six weeks post infection (**Figures 1**, **6A**). Furthermore, adult PBMCs samples also produced higher levels of IFNγ at T2 and T3, compared to T1, after TLR2 stimulation (**Figure 6B**), TLR4 (**Figure 6C**), and TLR7/8 (**Figure 6D**) stimulation. In addition, TLR2 and TLR4, but not TLR7/8, stimulation also resulted in higher production of IL-1β in T2 and T3 samples compared to T1 samples (**Figures 6B–D**). The response to iSARS-CoV-2 or TLR stimulating agents by PBMCs from PCR+ children collected at T2 did not differ from T1 (data not shown). Together, these data indicate that at 2 and 6 weeks post infection adult PBMCs increase their responsiveness to iSARS-CoV-2 or PRR stimuli, whereas children retained stable response at T2.

## Discussion

During the COVID-19 pandemic, children generally did not develop disease or when it occurred the severity, compared to adults, was relatively mild (5, 6). Several reports have argued that innate immune responses may determine the progression of COVID-19 (4, 7, 8, 28). In this study we showed that just a few days after primary infection, there are differences in the innate immune responses between PCR+ children and adults. The levels of SARS-CoV-2 Spike-specific IgG were also measured but no differences between children and adults were found.

SARS-CoV-2 has been described to suppress the innate arm of the immune responses during early stages of infection to evade the host directed anti-viral effect (29–31). In line with these observations, we observed that compared to the secretion profile of age matched unexposed healthy controls, adults showed early after testing PCR+ a reduced secretion of IL-8, which persisted for weeks. This was only observed in adults and not in children who on the contrary, showed enhanced innate immune response that did not change for two weeks after testing PCR+.

Previous studies have shown that the levels of IL-8 tended to decrease with more severe symptoms (32–34). Here we show that early after testing PCR+, PBMCs from adults with more severe COVID-19 symptoms produced less IL-8 upon iSARS-CoV-2 restimulation, corroborating previous findings. An interesting finding is that PBMC from PCR+ adults cultured in medium only secreted lower levels of IL-8 compared to PBMC from PCR+ children. Whether the observed differences are due to less production, downregulation, or upregulation of IL-8 receptors in the infected adults remains to be determined.

In our study, the levels of IL-6, IL-10, and CXCL10 production varied strongly for PBMCs from PCR+ adults and did not associate with symptom severity. PBMCs from PCR+ children did produce IL-10 after activation with iSARS-CoV-2, with higher levels in children with mild and moderate symptoms. Previous work showed that children resolved the innate immune response faster compared to adults, which could underly the difference in COVID-19 symptoms (10). Since the anti-inflammatory cytokine IL-10 did associate with symptoms severity in children, the exact role of this cytokine in controlling the immune response to SARS-CoV-2 remains to be determined.

When analyzing the innate cellular composition after iSARS-CoV-2 stimulation, no significant changes were observed in PCR+ children compared to healthy controls. Neeland MR et al. previously showed however that the overall monocyte count decreased during the acute phase of infection, which we are not able to deduce from our FACS analysis (35). What was clear from our findings is that in PCR+ adults the proportion of non-classical monocytes was significantly lower compared to the adult healthy controls and PCR+ children, which has been reported before (35). This decreased proportion of non-classical monocytes correlated with increased COVID-19 severity, a finding that has been previously reported by others (36, 37). A possible explanation for the smaller proportion could be that these non-classical monocytes migrate to tissues after infection. In fact, in a study using a single cell multi-omics approach, CD16+ blood monocytes were identified as potential source to replenish bronchoalveolar macrophages in the lungs of COVID-19 patients (38). Another interesting finding from our study is that the proportion of the non-classical monocytes correlated with the production of interferons, CXCL10 and IL-8. This fits with the notion that monocytes are a major source of CXCL10 in COVID-19 disease (39) and that non-classical monocytes after activation initiate an inflammatory response against viruses (40). In addition, our data show more production of CXCL10, IFNγ and IFNα2 by iSARS-CoV-2 stimulated PCR+ adult PBMCs at T2 and T3, which may in part can be explained by the observed increased proportion of non-classical and decreased proportion of classical monocytes at these time points. Altogether, the anti-viral response by PBMCs early after SARS-CoV-2 infection shows an important role for non-classical monocytes, chemokines CXCL10 and IL-8, as well as interferons.

The most relevant pattern recognition receptors recognizing SARS-CoV-2 are the RIG-I like receptors (RLR) (MDA5, RIG-I) and Toll like receptors (TLR) (TLR2, TLR4, TLR7 and TLR8) (27). Previous work has shown that myeloid cells from the nasal cavity in healthy and infected children express higher levels of RLR and TLRs compared to adults (12). This suggests that immune cells of children are primed for virus sensing resulting in stronger antiviral responses (9, 12). Our data, showed enhanced baseline cytokine/chemokine production by PBMCs from healthy and PCR+ children compared to adults. These differences were strengthened after iSARS-CoV-2 and PRR stimulation, indicating that this enhanced sensing and anti-viral state is also present systemically in children.

A limitation of our study is the use of an inactivated preparation of SARS-CoV-2. The heat treatment used may have denatured viral proteins, which may affect the measured immune response. Nevertheless, it is clear from our findings that stimulation with iSARS-CoV-2 did induce an anti-viral immune response.

In summary, we show that iSARS-COV-2 or TLR stimulation of PBMCS from individuals with a primary SARS-CoV-2 infection reveals a distinct innate immune profile for adults versus children. The lower levels of different chemokines/cytokines, including IL-8 and CXCL10, and a lower proportion of non-classical monocytes characterized the adult participants, particularly those with severe COVID-19. In contrast, the PCR+ children showed a clear inflammatory response and no or mild symptoms. The novelty of the data presented here reinforces the hypothesis that the quality of the innate immune response to SARS-CoV-2 is crucial for disease development and implicates an important role for PRR activation and their downstream signaling pathways.

## Data Availability

The raw data supporting the conclusions of this article will be made available by the authors, without undue reservation.
