# Supplementary material for: Early immune profiling reveals distinct inflammatory responses between children and adults few days after primary SARS-CoV-2 infection
Source: Front Immunol. 2024 Nov 18;15:1359993. doi: 10.3389/fimmu.2024.1359993 (PMC11609154; doi:10.3389/fimmu.2024.1359993)
Supplement: Supplementary file 1 [file DataSheet1.docx]

**Supplemental Materials**


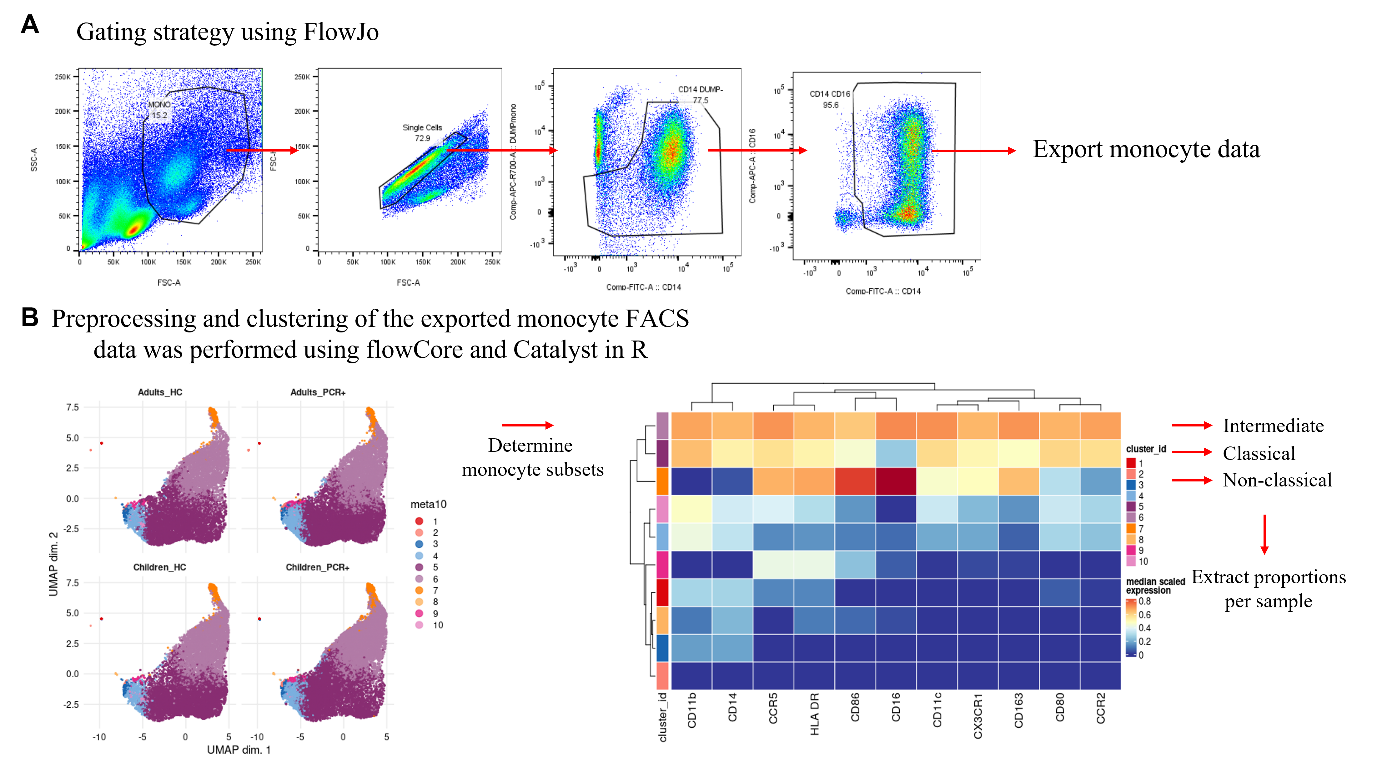


**Supplemental Figure 1. Gating strategy, preprocessing and clustering of FACS data.** Flowcytometric analysis were performed on iSARS-CoV-2 stimulated PBMCs to analyze the frequency of monocyte subset populations. Using FlowJo software bulk monocytes were gated and data was exported for further processing and clustering (A). Using the Flowcore and Catalyst packages in R we generated UMAPs per group (Healthy control adults and children, and PCR+ adults and children), after which the monocyte data was hierarchical clustered to determine the monocyte subsets (Classical (CD14+CD16-), Intermediate (CD14+CD16+), and non-classical (CD14lowCD16+). Lastly, the proportions of these subsets were extracted and plotted in Figure 4 and Supplemental Figure 2.





**Supplemental Figure 2. Changes in monocyte subset proportions in adults at different time points post infection.** Flow cytometric analysis of monocytes in iSARS-CoV-2 stimulated PBMCs of adults collected within <7days (T1), 2-3 weeks (T2), 4-6 weeks (T3) after testing PCR+. Proportions of classical monocytes (CD14+CD16-), intermediate monocytes (CD14+CD16+), and non-classical monocytes (CD14lowCD16+) among total monocytes in PBMCs. Kruskal-Wallis non-parametric test was used to compare different time points, after which Dunn’s multiple comparison test was executed. *p-adj<0.05, **p-adj<0.01, ***p-adj<0.001.





**Supplemental figure 3. Similar cytokine and chemokine profile induced by iSARS-CoV-2 in PBMCs from healthy control and recently infected children and adults.** Cytokine and chemokine levels were measured in culture supernatant from PBMCs after overnight stimulation with iSARS-CoV-2 (MOI=3). Kruskal-Wallis non-parametric test was used to compare healthy control (HC) to PCR+ individuals among children (▲) or adults (○), as well as HC or PCR+ children to their counterparts in adults, after which Dunn’s multiple comparison test was executed. Error bars indicate median value with 95% CI.

**

**

**Supplemental figure 4. Divergent cytokine and chemokine immune profile after TLR activation of PBMCs from healthy children compared to adults.** Cytokines and chemokines were measured in supernatant of PBMCs from healthy children (▲) and adults (○) after overnight stimulation of TLR2 with HKLM, TLR4 with LPS, or TLR7/8 with R848. Values are shown as log10 pg/ml. Multiple Mann-Whitney test with Bonferroni-Dunn multiple comparison test were performed to compared response by children and adults for each stimulation or mock. Error bars indicate median value with 95% CI. *p-adj<0.05. **p-adj<0.01
